# A Metabolism-Related Gene Landscape Predicts Prostate Cancer Recurrence and Treatment Response

**DOI:** 10.3389/fimmu.2022.837991

**Published:** 2022-03-10

**Authors:** Lijie Zhou, Ruixin Fan, Yongbo Luo, Cai Zhang, Donghui Jia, Rongli Wang, Youmiao Zeng, Mengda Ren, Kaixuan Du, Wenbang Pan, Jinjian Yang, Fengyan Tian, Chaohui Gu

**Affiliations:** ^1^ Department of Urology, First Affiliated Hospital of Zhengzhou University, Zhengzhou, China; ^2^ Department of Urology, Henan Institute of Urology and Zhengzhou Key Laboratory for Molecular Biology of Urological Tumor Research, First Affiliated Hospital of Zhengzhou University, Zhengzhou, China; ^3^ Department of Clinical Laboratory, The First Affiliated Hospital of Zhengzhou University, Zhengzhou, China; ^4^ Department of Obstetrics and Gynecology, First Affiliated Hospital, Xi’an Jiao tong University, Xi’an, China; ^5^ Department of Pediatrics, The First Affiliated Hospital of Zhengzhou University, Zhengzhou, China

**Keywords:** prostate cancer, metabolism, disease-free survival (DFS), immunotherapeutic response, drug sensitivity

## Abstract

**Background:**

Prostate cancer (PCa) is the most common malignant tumor in men. Although clinical treatments of PCa have made great progress in recent decades, once tolerance to treatments occurs, the disease progresses rapidly after recurrence. PCa exhibits a unique metabolic rewriting that changes from initial neoplasia to advanced neoplasia. However, systematic and comprehensive studies on the relationship of changes in the metabolic landscape of PCa with tumor recurrence and treatment response are lacking. We aimed to construct a metabolism-related gene landscape that predicts PCa recurrence and treatment response.

**Methods:**

In the present study, we used differentially expressed gene analysis, protein–protein interaction (PPI) networks, univariate and multivariate Cox regression, and least absolute shrinkage and selection operator (LASSO) regression to construct and verify a metabolism-related risk model (MRM) to predict the disease-free survival (DFS) and response to treatment for PCa patients.

**Results:**

The MRM predicted patient survival more accurately than the current clinical prognostic indicators. By using two independent PCa datasets (International Cancer Genome Consortium (ICGC) PCa and Taylor) and actual patients to test the model, we also confirmed that the metabolism-related risk score (MRS) was strongly related to PCa progression. Notably, patients in different MRS subgroups had significant differences in metabolic activity, mutant landscape, immune microenvironment, and drug sensitivity. Patients in the high-MRS group were more sensitive to immunotherapy and endocrine therapy, while patients in the low-MRS group were more sensitive to chemotherapy.

**Conclusions:**

We developed an MRM, which might act as a clinical feature to more accurately assess prognosis and guide the selection of appropriate treatment for PCa patients. It is promising for further application in clinical practice.

## Introduction

Prostate cancer (PCa) is the most common malignant tumor in men worldwide (1). Due to effective clinical interventions, including surgery, androgen deprivation therapy (ADT), and antiandrogen therapy, PCa has the highest 5-year survival rate (98%) among cancers. However, once recurrence occurs, including biochemical recurrence (BCR) and distant metastasis, which means that the patient is resistant to current treatment, the disease will progress rapidly without further effective intervention, eventually leading to death (2). The discovery of recurrent disease and the treatment of metastatic cancer are key issues for PCa patients (3). Although the system management of advanced PCa has made great progress in the last decade, there are still several problems. Docetaxel, the most common chemotherapy drug, has no survival benefit for PCa despite palliative responses in pain (4, 5). As second-generation antiandrogens, enzalutamide and abiraterone have been licensed for the treatment of castration-resistant PCa (CRPC); however, drug tolerance shortly follows treatment (6). Immunotherapy, including sipuleucel-T and ipilimumab, has emerged as a promising treatment for cancer. However, because PCa is a “cold” tumor with T-cell exhaustion, the response to single-agent checkpoint inhibition is limited (7, 8). Apart from that, precise biomarker therapies provide more possibilities for PCa treatment (1, 9). Combination therapy is a new trend in the treatment of advanced PCa, but multiple treatment options may lead to excessive medical treatment (10). Therefore, how to predict recurrence more easily and accurately, as well as to guide the selection of effective and sensitive treatments, is the focus of clinical research on PCa.

Metabolic reprogramming, considered a hallmark of cancer, has attracted increasing attention from researchers worldwide. It is well known that Warburg was the first person to characterize the aerobic glycolysis of tumor cells, named the Warburg effect. This phenomenon has been demonstrated in multiple solid tumors and is relevant to poor prognosis (11–13). With advances in biological sciences, tumor-related metabolic changes are more thoroughly understood. Tumor heterogeneity studies have also shown that different metabolic patterns occur even within the same tumor or tumor progression (14). Moreover, tumor microenvironment (TME), including endothelial cells, fibroblasts, and immune cells, consumes certain nutrients and then forces cancer cells to adapt by inducing nutrient scavenging mechanisms leading to cell proliferation (15). It has been recognized that the landscape of tumor metabolism is enormous and complicated. PCa also undergoes metabolic reprogramming and exhibits a unique metabolism that changes during initial neoplasia to advanced PCa (16, 17). Lipid metabolism and amino acid metabolism, rather than glycolysis, are the main energy supply for PCa. Metabolic reprogramming in PCa cells provides sufficient energy and important substances for tumor progression and drug resistance (18). Our previous study showed that mutual promotion occurs between the activation of the androgen receptor (AR) signaling pathway and lipid accumulation in PCa cells, which drives the progression to CRPC, and the abnormal accumulation of lipids is closely related to drug resistance to second-generation antiandrogens (19, 20). Metabolic rewiring in the TME, especially in immune cells, is closely related to immune evasion, tumor resistance, recurrence, and progression (21). Moreover, Luigi et al. reported that as the major intercellular substance of PCa, cancer-associated fibroblasts (CAFs) establish a metabolic symbiosis with PCa cells through lactate shuttling, resulting in tumor progression (22). However, most studies have focused only on the effects of certain gene changes on the metabolism of tumor cells or certain TME cells in PCa. Therefore, systematic and comprehensive exploration of the landscape of tumor metabolism will accelerate the development of the metabolic field in PCa and be more conducive to providing accurate clinical prognostic information and guiding treatment for PCa patients.

In the present study, we identified metabolism-related genes (MRGs) in PCa and constructed a prognostic indicator by Cox regression and least absolute shrinkage and selection operator (LASSO) regression analyses based on an MRG pair matrix and several PCa databases, which showed high accuracy in predicting recurrence and was associated with metabolic reprogramming, the immune microenvironment, and resistance to treatment.

## Results

### Identification and Analysis of Metabolism-Related Differentially Expressed Genes in Prostate Cancer

**Figure 1:**
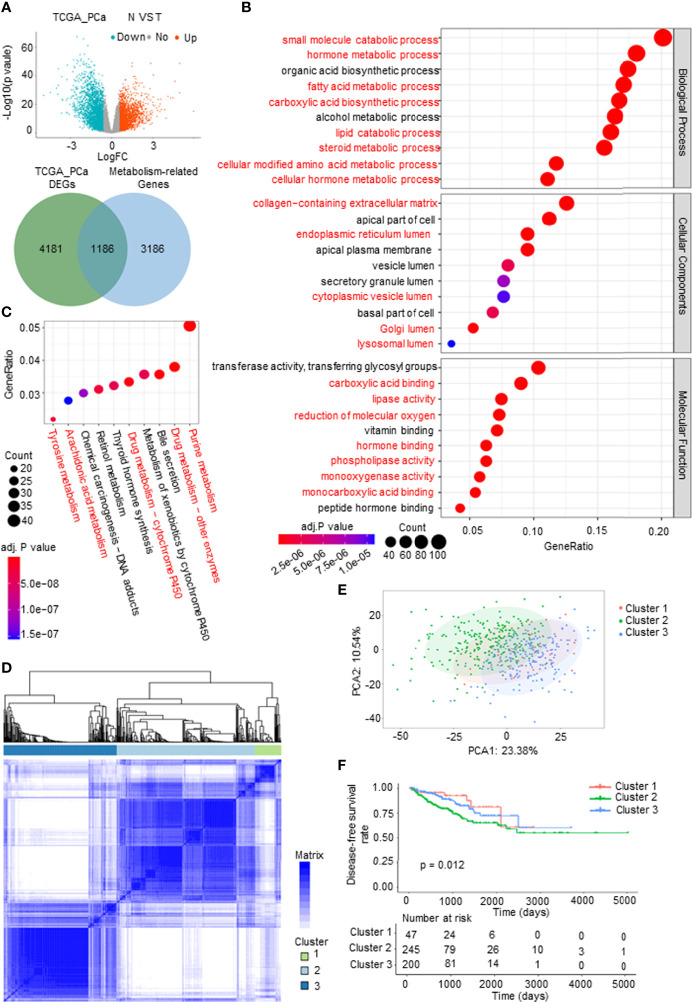
Identification and analysis of metabolism-related differentially expressed genes (mDEGs) in prostate cancer (PCa). **(A)** Volcano plot of the differentially expressed genes (DEGs) by comparing PCa tissues to normal prostate tissues from The Cancer Genome Atlas (TCGA) PCa database. Blue represents downregulated genes, and red represents upregulated genes in PCa. Venn diagram for the metabolism-related differentially genes (mDEGs). p < 0.05, |FC| > 1.5. **(B, C)** Bubble diagram of the top 10 terms in Gene Ontology (GO) and Kyoto Encyclopedia of Genes and Genomes (KEGG) enrichment analysis of the mDEGs. Adjusted p < 0.01, p < 0.05. **(D)** Consensus clustering of TCGA PCa cohorts based on the mDEGs. Consensus matrix for optimal k = 3. **(E)** Principal component analysis (PCA) of TCGA PCa database for optimal k = 3. **(F)** Kaplan–Meier analysis for disease-free survival (DFS) curves of patients in distinct clusters.

To systematically and comprehensively examine the tumor metabolic landscape at the gene level in PCa, we screened for metabolism-related differentially expressed genes (mDEGs) in The Cancer Genome Atlas (TCGA) PCa database. Comparing PCa to normal prostate tissues, 1,186 mDEGs were obtained on the basis of p < 0.05 and |Fold change| > 1.5 (**Figure 1A**). Gene Ontology (GO) and Kyoto Encyclopedia of Genes and Genomes (KEGG) enrichment analyses were implemented to investigate the potential functional implications of these genes and to obtain general knowledge of metabolic panorama changes in PCa. The top 10 enriched GO terms were as follows: BP included lipid metabolism, amino acid metabolism, hormone metabolism, and carboxylic acid biosynthesis; CP included extracellular matrix (ECM) and intracellular lumen (e.g., endoplasmic reticulum lumen and Golgi lumen); and MF included lipase activity, carboxylic acid binding, and hormone binding (**Figure 1B**). The KEGG enrichment analysis results showed that mDEGs were significantly associated with drug, arachidonic acid, tyrosine, and purine metabolism pathways (**Figure 1C**).

To investigate whether metabolic changes are related to the recurrence and progression of PCa, consensus clustering was performed to divide the tumor tissues into subgroups according to the expression of mDEGs (**Supplementary Figures 1A–C**). The following three distinct patterns were identified: 47 cases in Cluster 1, 245 cases in Cluster 2, and 200 cases in Cluster 3 (**Figures 1D, E**). The Kaplan–Meier (KM) survival analysis of disease-free survival (DFS; no recurrence/progression) with the three metabolic subtypes showed significant differences (**Figure 1F**). Collectively, these results illustrated that tumor metabolic reprogramming played an important role in PCa recurrence and progression.

### Screening Survival-Related Key Metabolism-Related Differentially Expressed Genes for Prostate Cancer

**Figure 2:**
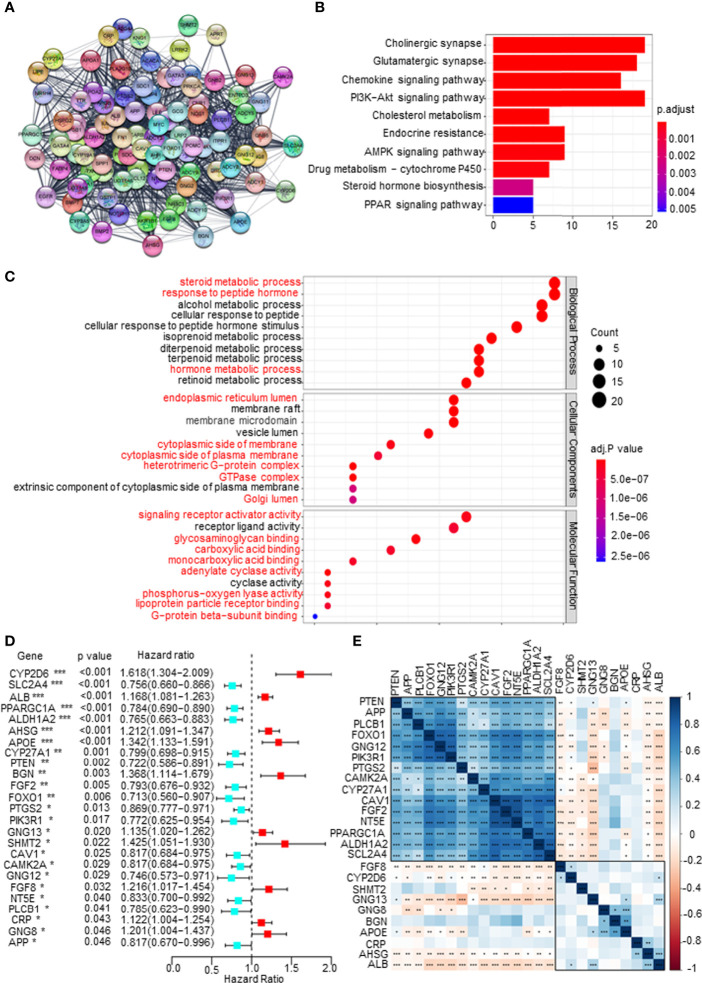
Screening disease-free survival (DFS)-related key metabolism-related differentially expressed genes (mDEGs) for prostate cancer (PCa). **(A)** Protein–protein interaction (PPI) network of the top 100 hub genes from these mDEGs. **(B, C)** Gene Ontology (GO) and Kyoto Encyclopedia of Genes and Genomes (KEGG) enrichment analysis for these hub genes. Adjusted p < 0.01 and p < 0.05 were considered significant. **(D)** Forest plot of the key mDEGs associated with DFS based on univariate Cox regression analysis for the top 100 hub genes, whose p < 0.05 identified the DFS-related key mDEGs. **(E)** The correlation of the DFS-related key mDEGs.

First, a protein–protein interaction (PPI) network was constructed *via* the STRING database to identify the hub genes of the 1,186 mDEGs. Based on the connection score, the top 100 hub genes were considered to play more important roles in the progression of PCa (**Figure 2A**). KEGG and GO analyses were executed. The results of KEGG analysis showed that key mDEGs were enriched in synapse pathways, energy metabolic pathways (PI3K-AKT, MAPK, and PARP), drug metabolism pathways, cholesterol metabolism pathways, endocrine resistance, and steroid hormone biosynthesis pathways, which were associated with PCa development (such as neuroendocrine transformation and CRPC progression). The top 10 enriched GO terms included steroid and hormone metabolic process, endoplasmic reticulum lumen, GTPase complex, and metabolite banding. The results are shown in **Figures 2B, C**, indicating the significant functions of key mDEGs in metabolism and PCa progression. Then using univariable Cox regression analysis, we screened the DFS-related mDEGs based on the top 100 hub genes. As shown in **Figure 2D**, 25 of the top 100 genes were markedly related to DFS based on p-value less than 0.05, including 10 genes that showed hazardous factors with hazard ratios (HRs) and 95% CI greater than 1, and 15 genes showed protective roles with HR and 95% CI less than 1. As shown in **Figure 2E**, most survival-related key mDEGs were correlated with each other, suggesting that the metabolic rearrangement in PCa is an overall change and not a single gene change.

### Construction of a Metabolism-Related Risk Model to Predict the Disease-Free Survival of Prostate Cancer Patients

**Figure 3:**
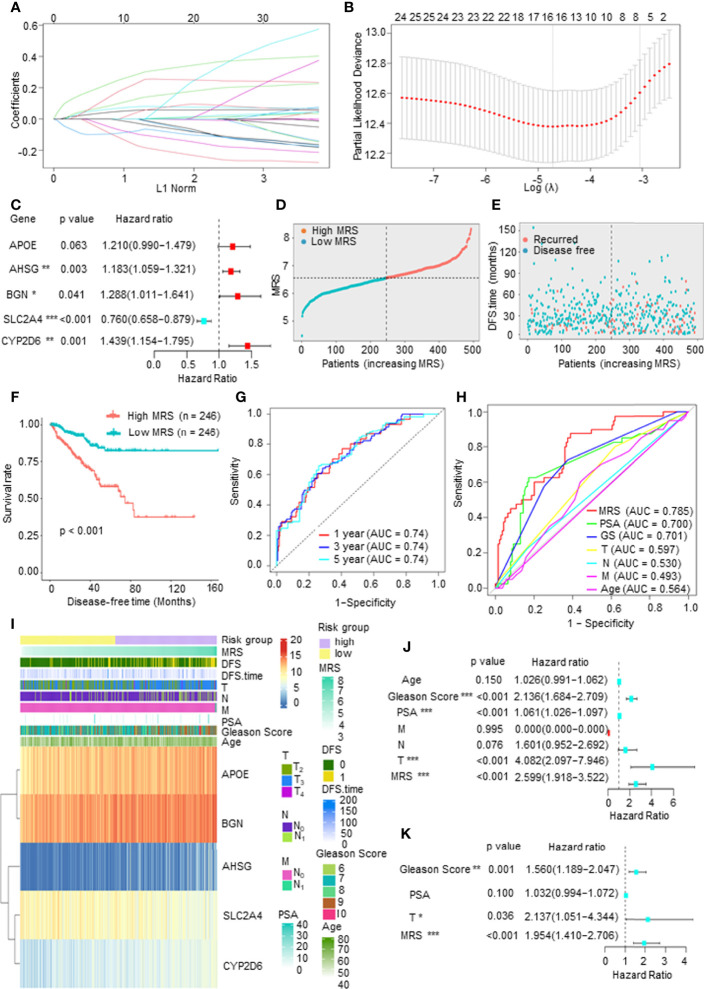
Construction of a metabolism-related risk model (MRM) to predict the disease-free survival (DFS) of prostate cancer (PCa) patients. **(A, B)** The least absolute shrinkage and selection operator (LASSO) Cox regression for the DFS-related key metabolism-related differentially expressed genes (mDEGs). **(C)** The multivariable Cox regression analysis of five genes based on cross-validation and the minimum partial likelihood deviance to further demonstrate the independent prognosis-related genes and obtain the genes index. **(D)** The distribution of risk scores in The Cancer Genome Atlas (TCGA) PCa based on median of metabolism-related risk score (MRS). Blue represents low-MRS subgroup, while red represents high-MRS subgroup. **(E)** The distribution of disease-free patients (blue) or recurrence (red) in subgroups. **(F)** Kaplan–Meier analysis for DFS curves of patients from TCGA PCa in low- or high-MRS subgroups. **(G)** Receiver operating characteristic (ROC) curves for predicting 1-, 3-, and 5-year DFS in patients from TCGA PCa database. **(H)** ROC analysis showed that the predictive accuracy of MRM was superior to other clinical features in TCGA PCa cohort. **(I)** The heatmap of five constituent genes of MRM, MRM characteristics, and clinical features in TCGA PCa database. **(J, K)** Univariate and multivariate Cox regression analyses of MRS and clinical features.

To establish a comprehensive and effective metabolism-related risk model (MRM) for prognosis, we performed LASSO Cox regression analysis for the DFS-related key mDEGs. After cross-validation, 5 genes (APOE, AHSG, BGN, SLC2A4, and CYP2D6) were highlighted by the minimum partial likelihood deviance (**Figures 3A, B**). Then, we carried out the multivariable Cox regression analysis to further demonstrate the independent prognosis-related genes and obtain the gene index. As shown in **Figure 3C**, APOE, AHSG, BGN, and CYP2D6 were the independent risk factors, and SLC2A4 was an independent protective factor. The MRM for prognosis was constructed based on the following formula: metabolism-related risk score (MRS) = 0.19 * the expression of APOE + 0.17 * the expression of AHSG + 0.25 * the expression of BGN + 0.36 * the expression of CYP2D6 − 0.27 * the expression of SLC2A4.

We then calculated the MRS according to this model and divided the 492 PCa patients into a high-MRS subgroup (n = 246) and a low MRS-subgroup (n = 246) based on the median MRS (**Figure 3D**). As shown in **Figures 3E, F**, the DFS rate of the high-MRS subgroup was obviously lower than that of the low-MRS subgroup, indicating that higher MRSs indicated a higher probability of recurrence. A receiver operating characteristic (ROC) curve demonstrated that MRM may act as a prognostic clinical feature. For 1-, 3-, and 5-year DFS, the area under the curve (AUC) values were 0.74, 0.74, and 0.74, respectively, suggesting that MRM had good sensitivity and specificity (**Figure 3G**). In addition, the ROC curve indicated that the predictive DFS accuracy of MRM was superior to other clinical parameters (Gleason score, prostate-specific antigen (PSA) level, and TNM stage and age) (**Figure 3H**). Besides, we explored the relationship among the MRS, clinical features, and the expression levels of the key mDEGs in TCGA PCa database. The heatmap in **Figure 3I** indicated that the expressions of APOE, BGN, AHSG, and CYP2D6 were increased in the high-MRS subgroup, while the expression of SLC2A4 was the opposite. We also found that the MRS was positively correlated with the Gleason score, T stage, N stage, and M stage but was not associated with PSA levels (**Figure 3I** and **Supplementary Figure 1D**). Moreover, univariate Cox regression demonstrated that the Gleason score, PAS level, T stage, and the MRS were closely related to DFS in PCa (**Figure 3J**), and multivariate Cox regression found that the p-value of the MRS (p < 0.001) was the lowest than other clinical features, suggesting that the MRS may be the most significant independent prognostic indicator of PCa (**Figure 3K**). Therefore, these findings suggested that MRM, a new feature, may be a better indicator to predict the DFS of PCa patients as compared to currently used prognostic factors.

### Validation of the Prognostic Value of Metabolism-Related Risk Model in Two Independent Prostate Cancer Cohorts and Real-World Study

**Figure 4:**
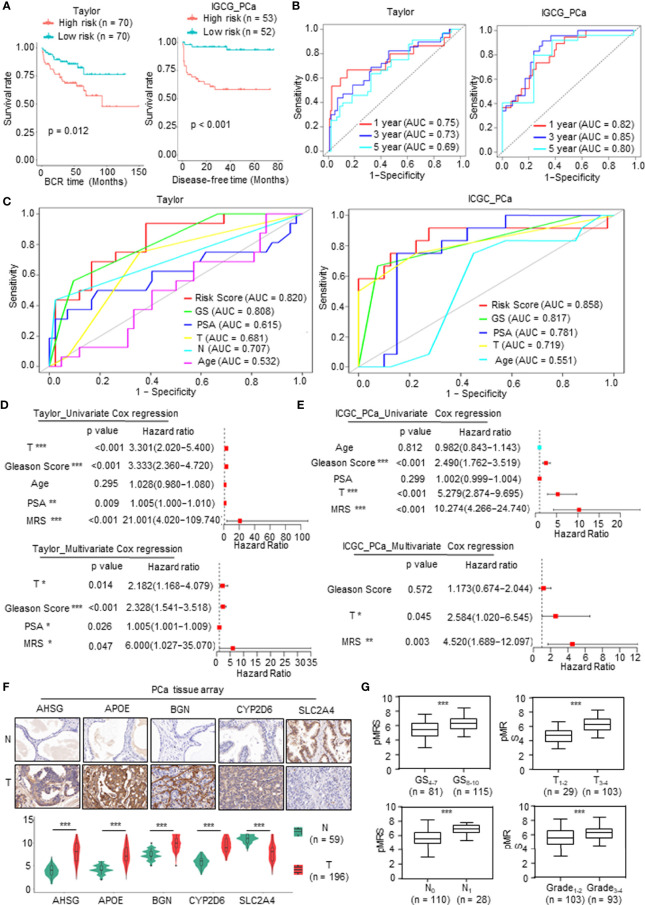
Validation of the prognostic value of metabolism-related risk model (MRM) in two independent prostate cancer (PCa) cohorts and real-world study. **(A)** Kaplan–Meier analysis for disease-free survival (DFS) curves of patients in low or high metabolism-related risk score (MRS) subgroups from two independent validation cohorts (Taylor and International Cancer Genome Consortium (ICGC) PCa). **(B)** Receiver operating characteristic (ROC) curves for predicting 1-, 3-, and 5-year DFS in patients from Taylor and ICGC PCa cohorts. **(C)** ROC analysis showed that the predictive accuracy of MRM in DFS was superior to other clinical features in Taylor and ICGC PCa cohorts. **(D, E)** Univariate and multivariate Cox regression analyses of MRS and clinical features in Taylor and ICGC PCa cohorts. **(F, G)** Immunohistochemistry (IHC) staining was performed to detect the key metabolism-related differentially expressed genes (mDEGs) (AHSG, APOE, BGN, SLC2A4, and CYP2D6) protein expression using PCa tissue arrays from 59 normal tissues and 196 tumor tissues. Representative images are shown. Statistical analysis of the immunoreactive score (IRS) scores of IHC staining. *p < 0.05; **p < 0.01; ***p < 0.001.

To validate the prognostic value of the model, we screened independent PCa databases, including DFS or recurrence data of PCa patients, and we found two available datasets, namely, the Taylor and International Cancer Genome Consortium (ICGC) PCa cohorts. As above, we calculated the MRS based on the metabolism-related risk model (MEM) equation. All patients were divided into the high-risk or low-risk subgroups based on the median MRS (**Supplementary Figure 2A**). Similar to the results derived from TCGA database, patients with higher MRS had shorter DFS time or BCR time, greater likelihood of recurrence, and worse prognosis (**Figure 4A** and **Supplementary Figure 2B**). The AUC values for 1-, 3-, and 5-year BCR in the Taylor PCa cohort were 0.75, 0.73, and 0.69, respectively, and those in ICGC PCa were 0.82, 0.85, and 0.80, respectively, demonstrating that the MRM may be a potential clinical feature that predicts DFS with high accuracy and reliability for PCa patients (**Figure 4B**). Moreover, as shown in **Figure 4C**, the ROC curve derived from the Taylor and ICGC PCa cohorts indicated that the predictive accuracy of MRM was superior to that of other clinical features. In addition, we also analyzed the relationship among the MRS, clinical features, and the expression levels of the key mDEGs in the Taylor and ICGC PCa database (**Supplementary Figure 2C**). The results of the Taylor and ICGC cohorts were consistent with those of TCGA. In Taylor, univariate and multivariate Cox regression analyses both showed that the Gleason score, PAS level, T stage, and MRS were closely related to DFS and could be independent prognostic indicators of PCa (**Figure 4D**). In the ICGC PCa cohort, univariate Cox regression analysis revealed that the Gleason score, T stage, and MRS were closely related to DFS; multivariate Cox regression analysis found that only the T stage and MRS were independent prognostic indicators of PCa, but the Gleason score was not an independent prognostic indicator of PCa (**Figure 4E**), which may be due to sample size, heterogeneity of data source, and composition. Thus, these data indicated that the MRM was the best independent predictor of the DFS or BCR in two independent PCa cohorts.

We next investigated the value of MRM in the real world using the tissue collected at the First Affiliated Hospital of Zhengzhou University. qRT-PCR was carried out to assess the mRNA expression of the DFS-related key mDEGs (APOE, AHSG, BGN, SLC2A4, and CYP2D6) in fresh PCa tissues and matched adjacent normal prostate tissues (n = 30). The MRS of the tissue was calculated based on the relative expression of genes. An immunohistochemistry (IHC) assay was utilized to measure the expression at the protein level, and the protein level of the MRS (pMRS) was obtained based on the immunoreactive score (IRS) of the DFS-related key mDEGs (APOE, AHSG, BGN, SLC2A4, and CYP2D6). As shown in **Supplementary Figure 3A** and **Figure 4F**, AHSG, APOE, BGN, and CYP2D6 were significantly upregulated in PCa tissues compared to normal prostate tissues, while SLC2A4 showed the opposite result. Further analysis revealed that MRS and pMRS had a close relationship with the Gleason score and T stage, and pMRS was positively correlated with N stage and Grade (**Supplementary Figure 3B** and **Figure 4G**).

In summary, these findings demonstrated that the MRS may be a promising prediction feature with high reliability and accuracy for PCa patients.

### The Molecular Function and Mechanism of Metabolism-Related Risk Score in Prostate Cancer

**Figure 5:**
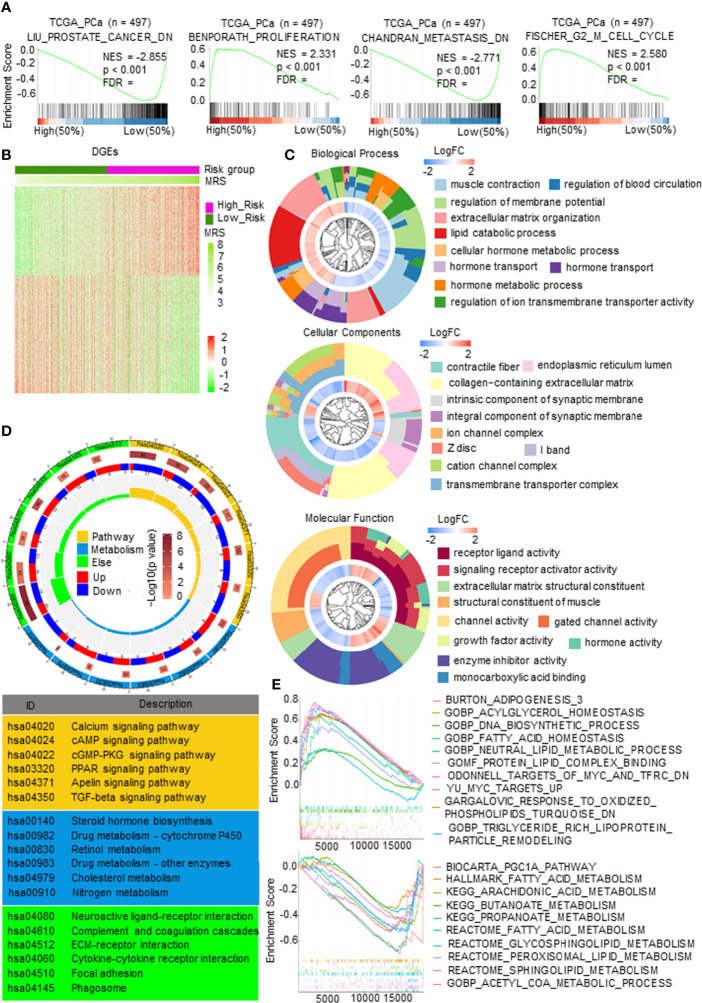
The molecular function and mechanism of metabolism-related risk model (MRM) in prostate cancer (PCa). **(A)** Gene set enrichment analysis (GSEA) of metabolism-related risk score (MRS) and PCa progression signaling pathways. p < 0.05 and false discovery rate (FDR) <0.05 were considered significant. **(B)** Heatmap of differentially expressed genes (DEGs) by comparing the expression between the high- and low-MRS groups. p < 0.05 and |FC| > 1.5. **(C)** Gene Ontology (GO) analysis for exploring molecular function and biological process involved in these DEGs. **(D)** Kyoto Encyclopedia of Genes and Genomes (KEGG) analysis revealed the major pathways in which these DEGs were involved in. **(E)** Metabolism-related, lipid metabolism-related gene sets enriched in high- or low-MRS subgroup (p < 0.05, FDR < 0.25).

First, gene set enrichment analysis (GSEA) was carried out to predict the gene set changes between the high- and low-MRS groups in the PCa TCGA cohort. The results revealed that the gene sets of the high-MRS samples were gathered in pathways related to proliferation and cell cycle, while the low-MRS samples were enriched in gene sets of genes downregulated in PCa and downregulated in metastatic tumors from the panel of patients with PCa (**Figure 5A**), suggesting that there was a significant difference in tumor growth and metastasis between MRM subgroups. We then analyzed gene mutations to further explore genetic differences in the MRM subgroups. Missense variations were the most common mutation type, and the high-MRS group had higher mutation counts than the low-MRS group. The top 10 genes with the highest mutation rates in the MRS subgroups are shown in **Supplementary Figure 4A**. The mutation rates of TP53, SPOP, TTN, FOXA1, and SYNE1 genes in the high MRS subtype were higher than those in the low MRS, while the mutation rates of the KMT2D and MUC16 genes in the high MRS subtype were lower than those in the low MRS subtype; the mutation of SPTA1, LRP1B, and KMT2C genes was more common in the high-MRS group, while the mutation of RYR2, ATM, and RP1 genes was more common in the low-MRS group.

Gene expression analysis was carried out and identified 1,258 genes by comparing the high and low-MRS groups (**Figure 5B**). Further research found that the DEGs were mostly enriched in multiple metabolic processes, transmembrane transporter complex, and enzyme activity (**Figure 5C**). KEGG analysis also demonstrated that these DEGs were closely related to pathways in metabolism, such as the PPAR signaling pathway, cAMP signaling pathway, steroid hormone biosynthesis, cholesterol metabolism, and drug metabolism (**Figure 5D**). **Figure 5E** and **Supplementary Figure 4B** show representative metabolic pathways derived from KEGG analysis, especially pathways in lipid metabolism and amino acid metabolism. The results revealed that the subgroups had different metabolic characteristics, as follows: the high-MRS group had more lipid synthesis and less lipid degradation than the low-MRS group, and the low-MRS group involved more amino acid metabolism and lipolysis than the high-MRS group. In addition, GO and KEGG enrichment analysis showed that the DEGs were also associated with synapses, ECM, and cytokines, suggesting that the MRM had a relationship with neuroendocrine transformation and that the subgroups had different microenvironments (**Figures 5C, D**).

### Immune Characteristics of Different Metabolism-Related Risk Score Subgroups

**Figure 6:**
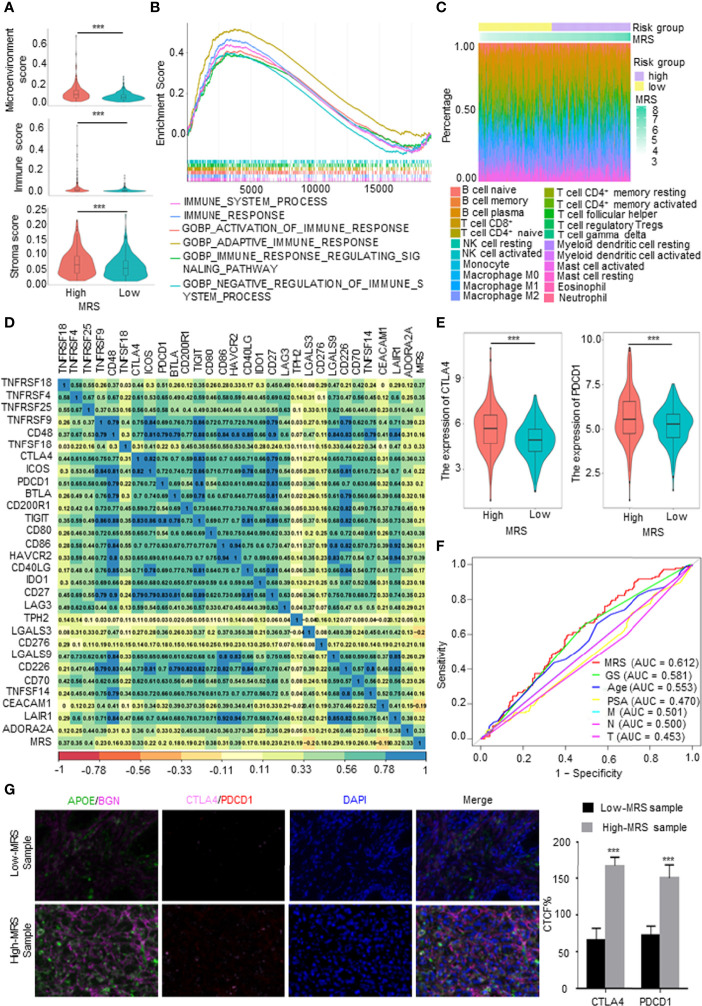
Immune characteristics of different metabolism-related risk score (MRS) subgroups. **(A)** Comparison of the microenvironment, immune, and stromal score in subgroups with high- and low-MRS. **(B)** Immune-related gene sets enriched in high-MRS subgroup [p < 0.05, false discovery rate (FDR) <0.25]. **(C)** The landscape of immune cells along with MRS subgroups from The Cancer Genome Atlas (TCGA) prostate cancer (PCa) patients. **(D)** Correlations between the MRS and classical immune checkpoints. p < 0.05 and |R^2^| > 0.15. **(E)** The expression of CTLA4 and PDCD1 in high- and low-MRS subgroups. **(F)** Receiver operating characteristic (ROC) analysis showed that the predictive accuracy of metabolism-related risk model (MRM) in response to immunotherapy was slightly superior to other clinical features in TCGA PCa cohorts. **(G)** Multiplex immunofluorescence (mIF) confirmed CTLA4 and PDCD1 expressions in the high-MRS subgroup were higher than those in the low-MRS subgroup. Quantified the corrected total cell fluorescence (CTCF) of these genes stained. ***p < 0.001.

The TME, which is the surrounding microenvironment of tumor cells, includes immune cells, surrounding blood vessels, fibroblasts, extracellular stroma, and various signaling molecules. First, we explored the relationship between the MRS and the TME. The MRS was positively correlated with the TME score, and the high-MRS group presented higher immune cell infiltration and stroma scores than the low-MRS group (**Figure 6A**). The gene sets of the high-MRS samples were enriched in immune response-related pathways (**Figure 6B**). The detailed immune cell regulatory pathways of GSEA are shown in **Supplementary Figure 5A**. Besides, consistent with the predicted results in **Figure 6A**, pathways related to ECM were highly associated with the high MRS (**Supplementary Figure 5B**). Subsequently, we further investigated the correlation between MRS and immune cell infiltration in PCa. We calculated the proportion of immune cells in PCa samples from TCGA database using the following six independent algorithms: CIBERSORT, xCell, quanTIseq, MCP-counter, EPIC, and ImmuCellAI. The characteristics of the immune landscape related to MRS are displayed in **Figure 6C**. We also found that the infiltration of B cells, CD4^+^ T cells, macrophages, dendritic cells, and CAFs were more abundant in the high-MRS subgroup, while CD8^+^ T cells and neutrophils were more abundant in the low-MRS subgroup (**Supplementary Figure 5C** and **Supplementary Table 1**). We screened and collected classical immune checkpoints, and then we applied those genes to define the immune and molecular functions between the different MRS groups. We found that the MRS was closely correlated with the expression of 30 immune checkpoints (p < 0.05, R > 0.15), including PDCD1, PDL1, and CTLA4 (**Figure 6D**). Notably, the expressions of CTLA4 and PDCD1 were significantly elevated in the high-MRS samples (**Figure 6E**), suggesting that immunotherapy may be a relatively effective treatment for these patients. Therefore, we predicted the response to immunotherapy in PCa patients derived from TCGA (**Supplementary Figure 5D**). In terms of predicting the response to immunotherapy, the AUC value of the MRM is the highest among clinical features (**Figure 6E**), indicating that the new feature, MRM, may be a potential indicator in predicting the immunotherapeutic response of PCa patients. Finally, we performed multiplex immunofluorescence (mIF) to detect the expression of the key MRGs and classical immune checkpoints and analyzed their correlation. Although immune checkpoint expression was relatively weak in PCa, CTLA4 and PDCD1 expressions in the high-MRS subgroup were both higher than those in the low-MRS subgroup (**Figure 6F**). As predicted, these results suggested that the high-MRS subgroup was more likely to benefit from immunotherapy than the low-MRS subgroup.

### Sensitivity to Drugs in Prostate Cancer Patients With Different Metabolism-Related Risk Score Subgroups

**Figure 7:**
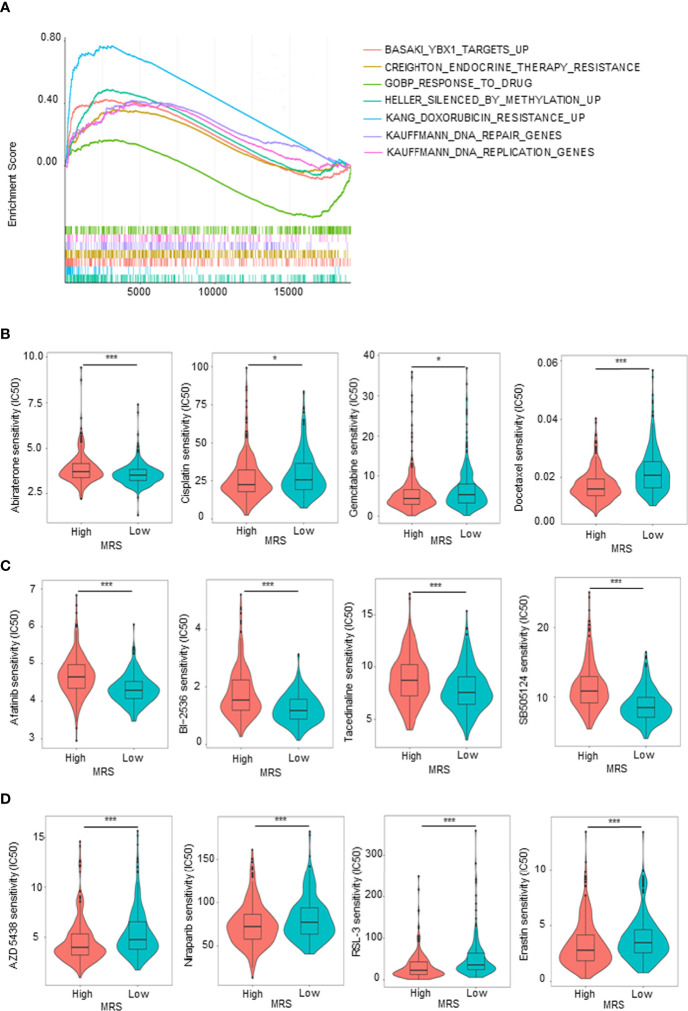
Sensitivity to drugs in prostate cancer (PCa) patients with different metabolism-related risk score (MRS) subgroups. **(A)** Gene set enrichment analysis (GSEA) showed the drug metabolism-related gene sets enriched in high- or low-MRS subgroup (p < 0.05, false discovery rate (FDR) <0.25). **(B)** Estimated sensitivity of current clinically preferred drugs for advanced PCa in patients with high and low MRS risk. **(C, D)** Predicting sensitivity of potential drugs for advanced PCa in patients with high and low MRS risk. *p < 0.05; ***p < 0.001.

Given that the GO and KEGG analyses (**Figures 5C, D**) suggested that MRS is involved in drug metabolism, hormone synthesis, and metabolism-related pathways, while antiandrogen therapy and chemotherapy are the dominant treatment for advanced PCa, we hypothesized that patients in different MRS subgroups have different sensitivities to drugs. We first analyzed in detail the pathways related to drug metabolism identified by KEGG enrichment analysis of the MRS subgroups. We found that pathways related to endocrine therapy resistance, DNA repair genes, silenced by methylation, and doxorubicin resistance were associated with high MRS, while genes related to drug response were positively correlated with low MRS (**Figure 7A**). Then, we compared the sensitivity of the high- and low-MRS groups to common anticancer drugs to guide treatment options for different PCa patients. In terms of standard drug selection, patients in the low-MRS subgroup were more sensitive to antiandrogen (abiraterone), while patients in the high-MRS group were more sensitive to chemotherapy (docetaxel and gemcitabine–cisplatin (GC) chemotherapy) (**Figure 7B**). In terms of recommended drug selection, patients in the low-MRS subgroup were more sensitive to EGFR inhibitors (e.g., afatinib), BI-2536 (PLK1 and BRD4), HDAC inhibitor (tacedinaline), and TGF-β receptor inhibitor (SB-505124), while patients in the high-MRS subgroup were more sensitive to CDK inhibitors (e.g., AZD 5438), PARP inhibitors (e.g., niraparib), and ferroptosis agonist (RSL3, erastin) (**Figures 7C, D**). Similar results are shown in **Supplementary Figures 6A–C** based on the Taylor database.

## Discussion

Cell metabolic reprogramming, an important hallmark of tumors, contributes to tumor initiation and progression. Accompanied by changes in intracellular and extracellular metabolites, metabolic reprogramming has a profound impact on gene expression, cellular heterogeneity, and the TME (12–15). Metabolic reprogramming also occurs in PCa and exhibits a unique metabolism that changes during initial neoplasia to advanced PCa (16, 17). Patients with PCa, an indolent tumor, have a long survival with effective clinical interventions; but PCa progresses rapidly once tumor recurrence occurs (23). Although there are many treatment options for advanced PCa, such as new endocrine therapy, immunotherapy, or chemotherapy, these treatments are ultimately not effective long enough to change the ultimate outcome of the disease—death (2, 24). Accumulating studies have demonstrated that metabolic rewriting in PCa is closely related to tumor progression, tumor recurrence, endocrine therapy tolerance, and immunotherapy nonresponse (17, 25, 26). Given that recurrence is a turning point in disease progression and treatment as well as that metabolism plays an important role in PCa, we collected all the MRGs based on Molecular Signatures Database (MSigDB) and several PCa datasets, systematically and comprehensively explored the landscape of tumor metabolism, and constructed a metabolism-related model based on DFS or relapse-free survival to provide accurate clinical prognostic information and guide treatment for PCa patients.

In this study, we first identified and analyzed mDEGs in TCGA PCa database, and these mDEGs were mainly enriched in lipid- and amino acid-related metabolic processes. Consistent with other studies, metabolic rearrangement in PCa occurs mainly in lipid and amino acid metabolism, rather than glucose metabolism (17). Notably, these mDEGs were also enriched in hormone metabolism and drug metabolism-related pathways. As we know, PCa is an androgen-dependent tumor (27). The results indicated that metabolic reprogramming in PCa involves not only metabolic reprogramming of energy substances but also changes in hormone and drug metabolism. Based on these DEGs, consensus clustering analysis found that patients could be divided into three subgroups, and there were significant differences in the DFS of the three subgroups. These findings indicated that metabolism in PCa is heterogeneous and that patients with different metabolic patterns have different prognoses.

Subsequently, through PPI network, univariable and multivariable Cox regression analyses, and LASSO Cox regression analysis, we screened 5 survival-related key mDEGs, including APOE (apolipoprotein E), AHSG (α2-HS-glycoprotein), SLC2A4 (solute carrier family 2 member 4), BGN (biglycan), and CYP2D6 (cytochrome P450 family 2 subfamily D member 6). These genes have been reported to be involved in metabolism or PCa progression previously. As we know, APOE is a key cholesterol regulatory protein (28). Ifere et al. reported that APOE influences aggressive behavior in PCa cells by deregulating cholesterol homeostasis (29), and Marco et al. demonstrated that the expression of APOE was directly correlated with the Gleason score, local and distant aggressiveness, and hormone independence in PCa (30). Fetuin-A, the protein product of AHSG gene, is a hepatokine and is known to be associated with insulin resistance and type 2 diabetes (31). Carol et al. reported that AHSG plays a role in tumor progression by interfering with the binding of TGF-β1 to colorectal cancer cell surface receptors (32). However, the role of AHSG in PCa is unclear. SLC2A4, also named GR-mediated glucose transporter 4 (GLUT4), is associated with glucose supply and metabolism, and it is upregulated with chronic Enz treatment (33, 34). CYP2D6, a drug-metabolizing cytochrome P450 enzyme, is a critical pharmacogene involved in the metabolism of approximately 25% of commonly used drugs, and altered CYP2D6 function has been concerned with reduced drug efficacy (35). It has been reported that abiraterone could inhibit the expression of several drug-metabolizing cytochrome P450 enzymes, including CYP2D6 (36, 37). Ying et al. have demonstrated that as a key modulator, BGN regulates the key molecular pathways of metabolism and brain function (38). Yuan et al. reported that BGN, a proteoglycan of the ECM, was identified as a fibroblast-specific biomarker of poorer prognosis in colorectal cancer (39). Frank et al. have demonstrated that BGN is upregulated in PCa and closely related to clinical features of PCa patients (40), but the mechanism is unclear. Compared to normal tissues, we found that APOE, AHSG, CYP2D6, and BGN were upregulated in PCa tissues, while SLC2A4 was downregulated. The specific mechanisms of these key genes in metabolism, immunotherapy, and drug reactivity of PCa remain unclear, which is one of the limitations of this study. We will continue to study them further in the future.

Based on these five genes, MRSs were calculated, and an MRM was ultimately constructed for the prognosis prediction of PCa patients. TCGA PCa database was used as the training set, and two independent databases, Taylor and ICGC PCa cohorts, were used as the validation set. Further analysis in both the training set and the validation set revealed that the MRS had a close relationship with the clinical features of PCa, especially the TNM stage and Gleason score, indicating that the progression of PCa is accompanied by changes in tumor metabolism. Survival analysis, ROC curve analysis, and univariate and multivariate Cox regression analyses manifested that this MRS is a credible and calculable independent prognostic indicator. Noteworthy, the MRS has the highest accuracy in predicting tumor prognosis compared to other clinical indicators, including the Gleason score and PSA value, which comprise the gold standard for predicting prognosis in the clinic. These findings suggested that MRM may be a promising clinical prognostic indicator of PCa. To explore the possibility of clinical transformation of MRM, we detected the mRNA level and protein level expression of APOE, AHSG, CYP2D6, SLC2A4, and BGN in clinical specimens collected in the real world by qRT-PCR and IHC, and then we calculated MRS of each patient according to the model formula. Although clinical recurrence information is lacking, retrospective studies have found that MRS was closely associated with the clinical features of those patients. It is worth noting that the training and validation cohorts mainly consisted of non-Asian participants, whereas the clinical specimens collected in the real world were all from Asia, which indicated that this model has strong universality. However, the lack of tumor recurrence data in the real world is the limitation of our study. In the future, we will closely follow up on the survival data of the sample we collected and continue the study.

Recent studies have demonstrated crosstalk between cellular metabolic writing and the remodeling of the TME (41, 42). Although most PCa patients have a poor response to immunotherapy, improving the immune response efficiency has been the focus of PCa immune research (7, 43). In the present study, we quantified tumor metabolism through the calculated risk score (MRS) based on the construction of the MRM, objectively revealing the correlation between the relatively global metabolic reprogramming and the immune microenvironment, which may guide the different treatment approaches of the two groups. For example, there were CD4^+^, CD8^+^, B cells, and macrophage cells infiltrated in the high-MRS subgroup. At the same time, the expression of most classical immune checkpoints, such as CTLA4 and PD-L1, was also upregulated in this cluster, which may restrict the antitumor function of these cytotoxic cells. Moreover, the feature of MRM could predict tumor immune response more effectively than other clinical characteristics, such as the Gleason score and PSA value. Therefore, quantifying tumor metabolism through the MRM may help to predict tumor immune responses and avoid immunosuppressive therapy in patients who do not respond immunologically.

PCa is an androgen-related tumor, whose progression is closely related to androgen metabolism (27, 44). Cholesterol esters are the precursors of androgen synthesis, and recent studies have demonstrated that lipid metabolism reprogramming is closely related to endocrine therapy resistance in PCa (45). Notably, in the present study, we found that the high-MRS subgroup was more sensitive to abiraterone, a second-generation antiandrogen. Besides, chemotherapy is one of the options for treating advanced PCa. Drug metabolism is also a type of metabolism. It is well known that drug metabolism is closely related to chemotherapy resistance. In this study, we revealed that the low-MRS subgroup was more sensitive to classical chemotherapy agents, docetaxel and the GC regimens. Overall, we found that patients in the high-risk group were relatively sensitive to immunotherapy and endocrine therapy, while patients in the low-risk group were sensitive to chemotherapy. With the increasing popularity of molecular targeted therapy for cancer, we also predicted the sensitivity of different subgroups to other common molecular targeted drugs, so as to more accurately select sensitive targeted drugs according to the different tumor metabolism. These findings suggest the potential for the future application of the MRM in clinical guidance.

Tumor metabolic reprogramming is one of the characteristics of the tumor, which has attracted more and more attention. Our study identified the key mDEGs, presented a metabolic landscape, and constructed a MEM that systematically researches the relationship between tumor metabolic reprogramming, tumor recurrence, and the response to treatment (including immunotherapy, endocrine therapy, and chemotherapy) in PCa. There are more and more treatment options for advanced PCa. Choosing a more accurate therapeutic regimen according to the expression levels of key genes may be a direction of PCa treatment research in the future. We will further apply this model to clinical practice to test its effectiveness and hope that it could provide guidance for clinical treatment selection.

In conclusion, the present study identified the key mDEGs, presented a metabolic landscape, and constructed a MEM that exhibited high diagnostic accuracy in predicting DFS of PCa patients as well as predicting response to treatment. We hope that the utility of the constructed MRM can also be validated by additional clinical studies in the future.

## Methods

### Data Acquisition

TCGA PCa database was downloaded from the UCSC Xena website (http://xena.ucsc.edu/) as the training set, and the two independent PCa datasets: ICGC PCa (DKFZ, Cancer Cell 2018) and Taylor (MSKCC, Cancer Cell 2010) were downloaded from cBioPortal for Cancer Genomics (http://www.cbioportal.org/) as the training set (46, 47). The MRGs were searched and downloaded from Molecular Signatures Database (MSigDB, http://www.gsea-msigdb.org/gsea/msigdb/index.jsp). Immune checkpoints were collected from the literature.

### Differentially Expressed Gene Screening and Plot

Using R “limma” (Version 3.42.2) package, we screened the DEGs with p < 0.05, |Fold change| > 1.5 as the conditions (48). The volcano map or heatmap is performed with R “ggplot2” to show DEGs. A Venn diagram is used to show the intersection of DEGs and MRGs to identify mDEGs.

### Gene Ontology Analysis

GO analysis was implemented with R “clusterProfiler” (Version 3.14.3) package to further explore the possible functions of these DEGs (49). Adjusted p < 0.01 and p < 0.05 were considered statistically significant.

### Kyoto Encyclopedia of Genes and Genomes Analysis

KEGG analysis was carried out with R “clusterProfiler” (Version 3.14.3) and “enrichplot” package to look for the potential signaling pathway that DEGs or MRS is involved in (49). We defined p < 0.05 and false discovery rate (FDR) <0.25 as the cutoff criteria.

### Unsupervised Clustering Analysis

We performed hierarchical consistent cluster analysis using the R package ConsensusClusterPlus (50). The optimal number of clusters was determined through the consistent clustering algorithm, and the number of repetitions was set to 1,000 to ensure the stability of clusters.

### Protein–Protein Interaction Network Construction

We constructed a PPI network of mDEGs using the STRING online database (Version 10.0). The results were further imported into Cytoscape software (Version 3.6.1) to calculate the degree rank of hub genes using CytoHubba plug-in, and the top 100 hub genes were selected for future analysis (51).

### Construction and Validation of the Metabolism-Related Model to Evaluate the Metabolism-Related Risk Score

Univariate Cox regression was performed to screen DFS-related mDEGs (p < 0.05), followed by LASSO regression. With HR and 95% CI < 1, the gene was considered as a protective gene, while it was a risk gene with HR and 95% CI > 1. The correlation of each gene expression was displayed by the R “corrplot” package. Then, LASSO Cox regression was performed with the R “glmnet” package to avoid overfitting of recurrence features and narrow the range of genes predicting DFS (52). The key genes identified by LASSO regression were evaluated further by incorporating mDEGs into multiple Cox regression analyses. The MRM was constructed by weighting the estimated Cox regression coefficients. The model can be expressed as MRS = ∑ (βi × Expi), where the βi coefficient and Expi subscale represent the coefficient and normalized gene expression level, respectively. PCa samples were divided into low- and high-MRS subgroups according to the median MRS as the cutoff point.

The KM survival analysis was executed to estimate the difference in recurrence rates between the two risk subgroups. In addition, using the R “Survival ROC” package, ROC curves were applied to evaluate the specificity and sensitivity of the MRM based on the AUC. Then, we executed univariate and multivariate Cox regression analyses in order to validate the independent prognostic value of this model. Finally, the role of the MRS was further validated in two independent PCa validation sets (Taylor and ICGC PCa cohorts).

### Gene Mutation Analysis

Genetic alteration information was obtained from the cBioPortal database, and the number and quality of mutations in the two MRS subgroups were analyzed using the R “Maftools” package (53).

### Comprehensive Analysis of Molecular and Immune Infiltration Characteristics of Different Metabolism-Related Risk Score Subgroups

Based on the RNA-seq dataset of TCGA database PRAD, we evaluated the immune infiltration characteristics of PCa by seven online tools: CIBERSORT, TIMER, xCell, quanTIseq, MCP-counter, ImmuCellAI, and EPIC. Then, we compared the immune cells between the two MRS subgroups. Further, we carried out correlation analysis by the R “corrplot” package to assess the relationship between relapse risk scores and immune checkpoints. To assess the value of the MRM in prognostic immunotherapy response, the online tool TIDE (Tumor Immune Dysfunction and Exclusion, HTTP://TIDE.dfci.harvard.edu/) was used to perform immune checkpoint inhibitor response of each patient.

### Prediction of Drug Sensitivity in PRAD Between Different Metabolism-Related Risk Score Groups

Based on Genomics of Drug Sensitivity in Cancer (GDSC) and Cancer Therapeutics Response Portal (CTRP), we applied the R “oncoPredict” package to predict clinical response to multiple chemotherapy drugs (54). We compared the difference in 50% inhibitory concentration (IC50) between the high- and low-MRS subgroups.

### Human Samples

We obtained patients’ consent and approval from the Institutional Research Ethics Committee, then collected fresh PCa tissues and matched adjacent normal tissues from the First Affiliated Hospital of Zhengzhou University, and stored them at −80°C. Paired PCa and adjacent normal paraffin tissue sections were purchased from Xi’an ZK Biotech (M261601, M079Pr01) and Shanghai Outdo Biotech (HProA150CS01), and the clinical information was directly provided by companies.

### Quantitative Real-Time PCR Assay

We executed qRT-PCR assay according to the methods described previously (19). In brief, we extracted total RNA from samples using the TRIzol reagent (Thermo, Waltham, MA, USA). Then, we performed reverse transcription using The RevertAid First Strand cDNA Synthesis kit (TaKaRa, Dalian, China). Using the SYBR Green mix (TaKaRa), we carried out qRT-PCR on the StepOne Plus real-time PCR system (Life Technologies, Carlsbad, CA, USA). The primers sequences were as follows: APOE, Forward: 5′-GTTGCTGGTCACATTCCTGG-3′, Reverse: 5′-GCAGGTAATCCCAAAAGCGAC-3′; AHSG, Forward: 5′-TCCTTGGGGATACAAACACACC-3′, Reverse: 5′-TACCACGGAAAACTTGCCATC-3′; BGN, Forward: 5′-CAGTGGCTTTGAACCTGGAG-3′, Reverse: 5′-GGGAGGTCTTTGGGGATGC-3′; SLC2A4, Forward: 5′-TGGGCGGCATGATTTCCTC-3′, Reverse: 5′-GCCAGGACATTGTTGACCAG-3′; CYP2D6, Forward: 5′-TGGCAAGGTCCTACGCTTC-3′, Reverse: 5′-GCCACCACTATGCACAGGTT-3′; β-actin, Forward: 5′-CATGTACGTTGCTATCCAGGC-3′, Reverse: 5′-CTCCTTAATGTCACGCACGAT-3′.

### Immunohistochemical Staining Assay

IHC assay was carried out according to the methods described previously (20). To ensure the consistency of the analysis, all IHC assays were performed using the same type of tissue chips. The primary antibodies included anti-APOE (1:800, ab52607, Abcam, Cambridge, UK), anti-AHSG (1:1,000, ab187051, Abcam), anti-BGN (1:5,000, ab209234, Abcam), anti-SLC2A4 (1:500, 66846-1-Ig, ProteinTech, Chicago, IL, USA), and anti-CYP2D6 (1:500, ab185625, Abcam). We evaluated the expression of protein levels in the tissue sections based on the IRS: 0–1 indicating negative staining, 2–3 indicating mild staining, 4–8 indicating moderate staining, and 9–12 indicating strong positive staining.

### Multiplex Immunofluorescence Staining Assay

mIF assay was implemented according to the methods described previously (55). The primary antibodies included anti-APOE (1:50, ab52607, Abcam), anti-BGN (1:50, ab209234, Abcam), anti-CTLA4 (1:200, bs-1179R, Bioss, Woburn, MA, USA), and anti-PDCD1 (1:100, ab214421, Abcam). Using ImageJ software, we evaluated the expression of protein levels in the tissue sections on the basis of the corrected total cell fluorescence (CTCF).

### Statistical Analyses

All data visualization and statistical analysis were accomplished by R software (Version 4.1.2). Continuous variables between the two risk groups were compared *via* independent t-tests. Categorical data were carried out by the chi-square test. The MRS between different TIDE immunotherapy response groups was compared based on the Wilcoxon test. Pearson’s correlation coefficient is used to measure the correlation between two continuous variables. Univariate survival analysis was implemented *via* the KM method and the log-rank test. Multivariate survival analysis was carried out by the Cox regression model. p-Value <0.05 was considered statistically significant.

## Data Availability Statement

The original contributions presented in the study are included in the article/**Supplementary Material**. Further inquiries can be directed to the corresponding authors.

## Author Contributions

CG, LZ, and FT conceived the idea. LZ and RF conceived and designed the experiments. LZ, RF, and YL performed the experiments. DJ, YZ, MR, KD, WP, and JY helped to obtain clinical samples and matched information. LZ, RF, CZ, and RW analyzed the data. LZ and CG wrote the manuscript. All authors reviewed and approved the manuscript.

## Funding

This work was supported by the Natural Science Foundation of China (NO. 82173294); the Training Program for Middle-aged and Young Discipline Leaders of Health of Henan Province (NO. 202104); the Key Program Jointly Built by Henan Province and the Ministry of Medical Science and Technology (NO. SBGJ202102127 and SBGJ202102095); the Training Program of Young and Middle-aged Health Science and Technology Innovation Excellent Youth (NO. YXKC2021033); the Key Program of the Higher Education Institutions of Henan Province (NO. 20A320032); the Joint Construction Program of Medical Education and Research of Henan Province (NO. Wjlx2020054); the Program of International Training of High-level Talents of Henan Province (NO. 202207); Basic Research Incubation Program for Young Teachers of Zhengzhou University (NO. JC21854035).

## Conflict of Interest

The authors declare that the research was conducted in the absence of any commercial or financial relationships that could be construed as a potential conflict of interest.

## Publisher’s Note

All claims expressed in this article are solely those of the authors and do not necessarily represent those of their affiliated organizations, or those of the publisher, the editors and the reviewers. Any product that may be evaluated in this article, or claim that may be made by its manufacturer, is not guaranteed or endorsed by the publisher.
