# Supplementary material for: A Metabolism-Related Gene Landscape Predicts Prostate Cancer Recurrence and Treatment Response
Source: Front Immunol. 2022 Mar 10;13:837991. doi: 10.3389/fimmu.2022.837991 (PMC8960425; doi:10.3389/fimmu.2022.837991)
Supplement: Supplementary file 1 [file DataSheet_1.pdf]

SFig. 1

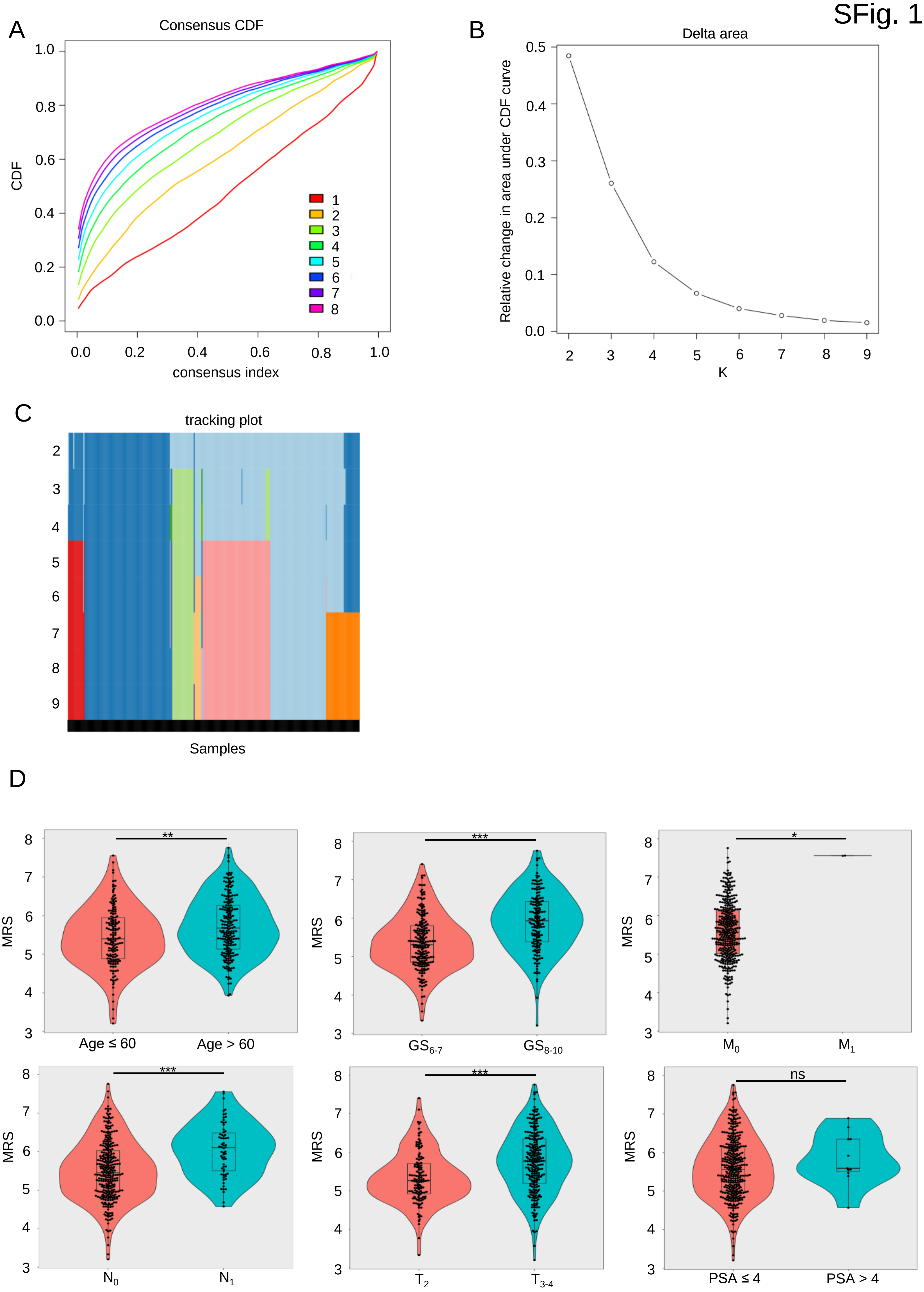

A
Consensus CDF
CDF
consensus index
1.0
0.8
0.6
0.4
0.2
0.0
1
2
3
4
5
6
7
8
B
Delta area
Relative change in area under CDF curve
K
0.5
0.4
0.3
0.2
0.1
0.0
2
3
4
5
6
7
8
9
C
tracking plot
Samples
D
MRS
Age ≤ 60
Age > 60
**
GS6-7
GS8-10
***
M0
M1
*
N0
N1
***
T2
T3-4
***
PSA ≤ 4
PSA > 4
ns

SFig. 2

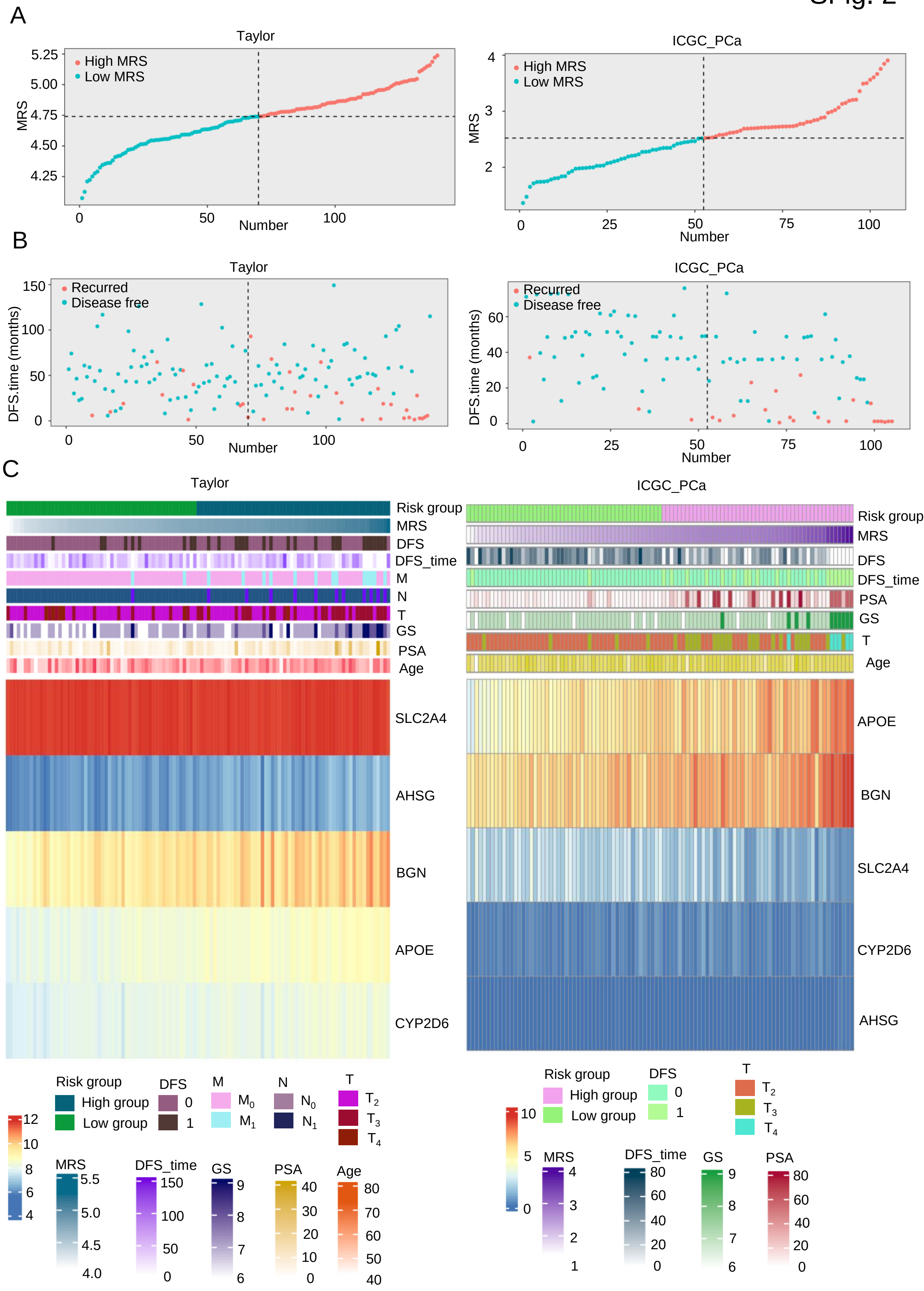

A
Taylor
High MRS
Low MRS
MRS
Number
ICGC_PCa
B
Recurred
Disease free
DFS.time (months)
C
Risk group
MRS
DFS
DFS_time
M
N
T
GS
PSA
Age
SLC2A4
AHSG
BGN
APOE
CYP2D6
High group
Low group

SFig. 3

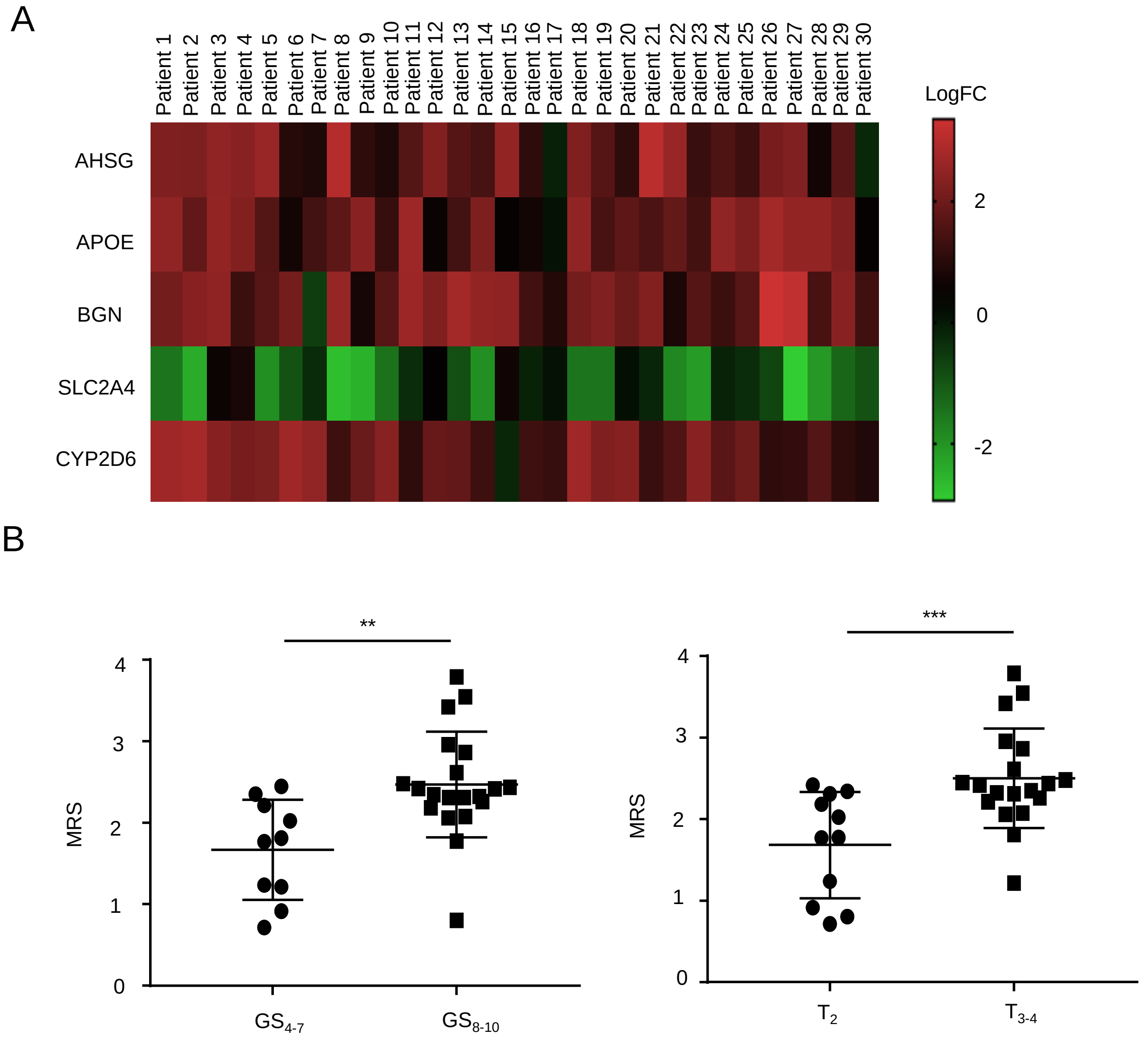
A
Patient 1
Patient 2
Patient 3
Patient 4
Patient 5
Patient 6
Patient 7
Patient 8
Patient 9
Patient 10
Patient 11
Patient 12
Patient 13
Patient 14
Patient 15
Patient 16
Patient 17
Patient 18
Patient 19
Patient 20
Patient 21
Patient 22
Patient 23
Patient 24
Patient 25
Patient 26
Patient 27
Patient 28
Patient 29
Patient 30
AHSG
APOE
BGN
SLC2A4
CYP2D6
LogFC
2
0
-2
B
**
***
MRS
0
1
2
3
4
GS4-7
GS8-10
T2
T3-4

SFig. 4

A

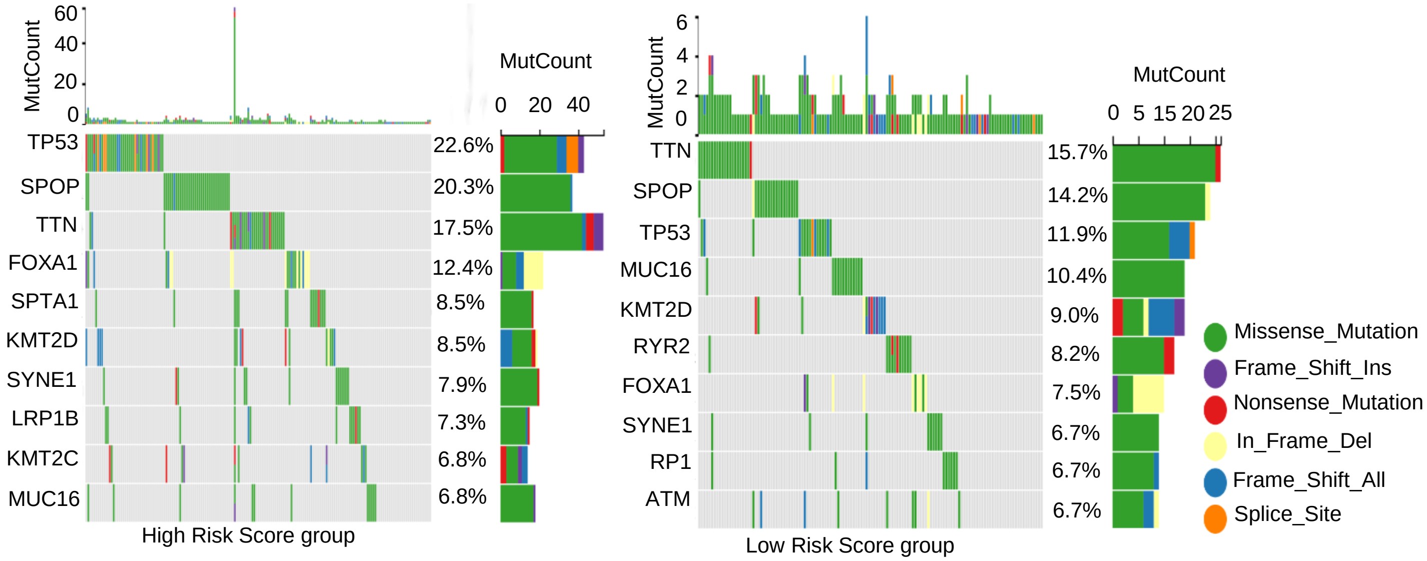

MutCount
60
40
20
0
MutCount
0 20 40
TP53 22.6%
SPOP 20.3%
TTN 17.5%
FOXA1 12.4%
SPTA1 8.5%
KMT2D 8.5%
SYNE1 7.9%
LRP1B 7.3%
KMT2C 6.8%
MUC16 6.8%
High Risk Score group
MutCount
6
4
2
0
MutCount
0 5 15 20 25
TTN 15.7%
SPOP 14.2%
TP53 11.9%
MUC16 10.4%
KMT2D 9.0%
RYR2 8.2%
FOXA1 7.5%
SYNE1 6.7%
RP1 6.7%
ATM 6.7%
Low Risk Score group
Missense_Mutation
Frame_Shift_Ins
Nonsense_Mutation
In_Frame_Del
Frame_Shift_All
Splice_Site

B

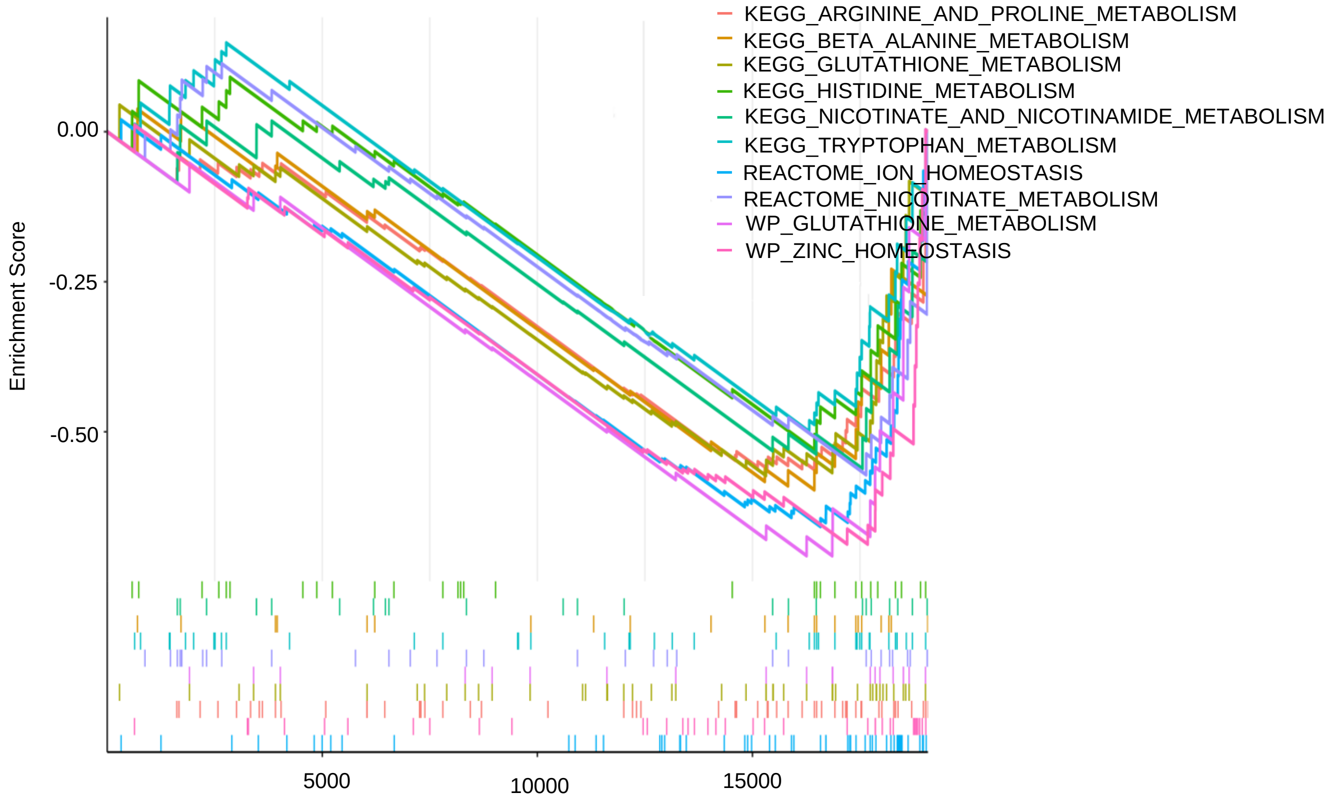

KEGG_ARGININE_AND_PROLINE_METABOLISM
KEGG_BETA_ALANINE_METABOLISM
KEGG_GLUTATHIONE_METABOLISM
KEGG_HISTIDINE_METABOLISM
KEGG_NICOTINATE_AND_NICOTINAMIDE_METABOLISM
KEGG_TRYPTOPHAN_METABOLISM
REACTOME_ION_HOMEOSTASIS
REACTOME_NICOTINATE_METABOLISM
WP_GLUTATHIONE_METABOLISM
WP_ZINC_HOMEOSTASIS
Enrichment Score
0.00
-0.25
-0.50
5000
10000
15000

SFig. 5

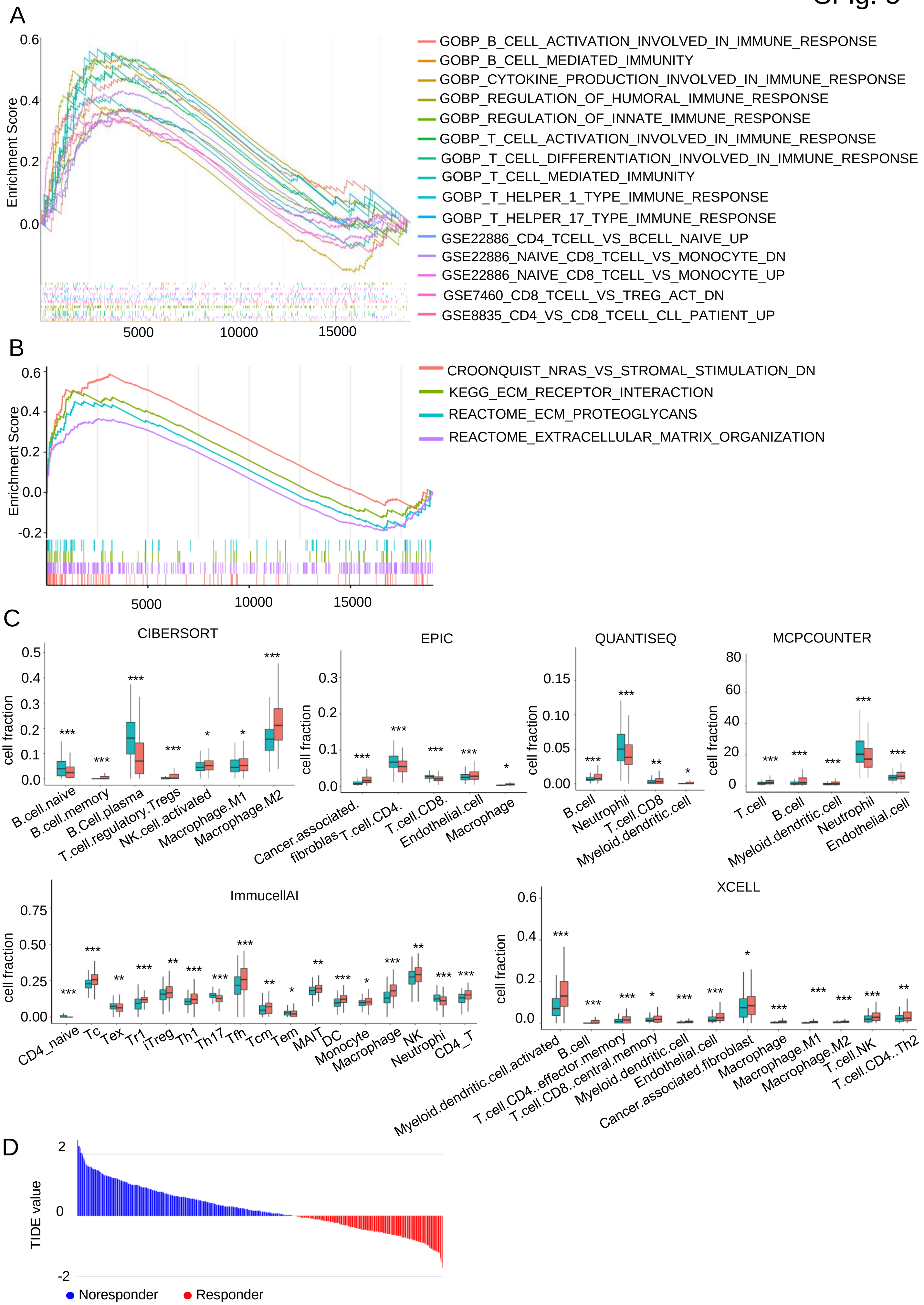

A
Enrichment Score
GOBP_B_CELL_ACTIVATION_INVOLVED_IN_IMMUNE_RESPONSE
GOBP_B_CELL_MEDIATED_IMMUNITY
GOBP_CYTOKINE_PRODUCTION_INVOLVED_IN_IMMUNE_RESPONSE
GOBP_REGULATION_OF_HUMORAL_IMMUNE_RESPONSE
GOBP_REGULATION_OF_INNATE_IMMUNE_RESPONSE
GOBP_T_CELL_ACTIVATION_INVOLVED_IN_IMMUNE_RESPONSE
GOBP_T_CELL_DIFFERENTIATION_INVOLVED_IN_IMMUNE_RESPONSE
GOBP_T_CELL_MEDIATED_IMMUNITY
GOBP_T_HELPER_1_TYPE_IMMUNE_RESPONSE
GOBP_T_HELPER_17_TYPE_IMMUNE_RESPONSE
GSE22886_CD4_TCELL_VS_BCELL_NAIVE_UP
GSE22886_NAIVE_CD8_TCELL_VS_MONOCYTE_DN
GSE22886_NAIVE_CD8_TCELL_VS_MONOCYTE_UP
GSE7460_CD8_TCELL_VS_TREG_ACT_DN
GSE8835_CD4_VS_CD8_TCELL_CLL_PATIENT_UP
B
Enrichment Score
CROONQUIST_NRAS_VS_STROMAL_STIMULATION_DN
KEGG_ECM_RECEPTOR_INTERACTION
REACTOME_ECM_PROTEOGLYCANS
REACTOME_EXTRACELLULAR_MATRIX_ORGANIZATION
C
CIBERSORT
EPIC
QUANTISEQ
MCPCOUNTER
cell fraction
B.cell.naive
B.cell.memory
B.Cell.plasma
T.cell.regulatory.Tregs
NK.cell.activated
Macrophage.M1
Macrophage.M2
Cancer.associated.fibroblas
T.cell.CD4.
T.cell.CD8.
Endothelial.cell
Macrophage
B.cell
Neutrophil
T.cell.CD8
Myeloid.dendritic.cell
T.cell
ImmucellAI
XCELL
CD4_naive
Tc
Tex
Tr1
iTreg
Th1
Th17
Tfh
Tcm
Tem
MAIT
DC
Monocyte
Macrophage
NK
Neutrophi
CD4_T
Myeloid.dendritic.cell.activated
T.cell.CD4..effector.memory
T.cell.CD8..central.memory
Endothelial.cell
Cancer.associated.fibroblast
Macrophage.M1
Macrophage.M2
T.cell.NK
T.cell.CD4..Th2
D
TIDE value
Noresponder
Responder

SFig. 6

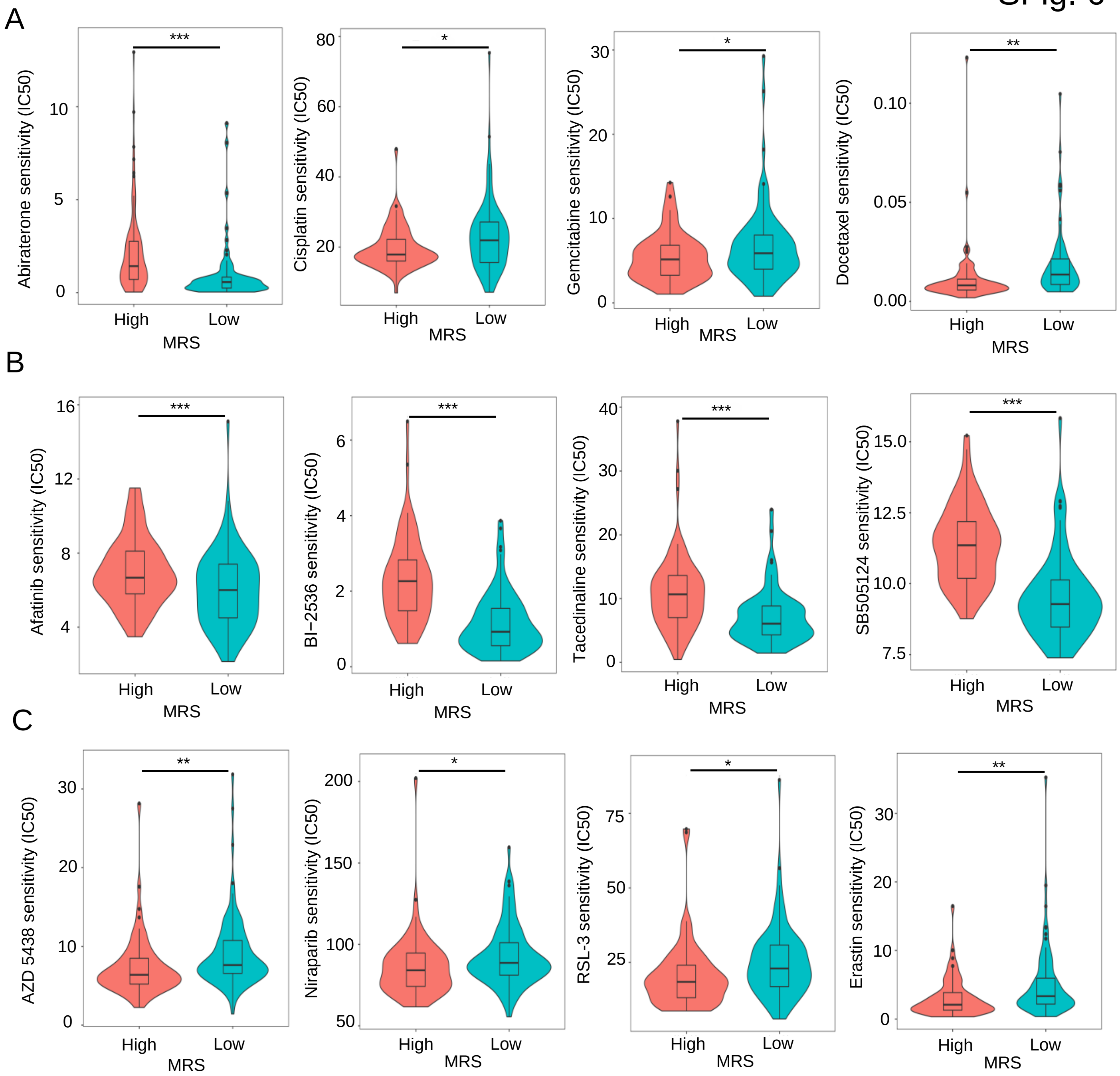
A
***
Abiraterone sensitivity (IC50)
High
Low
MRS
*
Cisplatin sensitivity (IC50)
High
Low
MRS
*
Gemcitabine sensitivity (IC50)
High
Low
MRS
**
Docetaxel sensitivity (IC50)
High
Low
MRS
B
***
Afatinib sensitivity (IC50)
High
Low
MRS
***
BI−2536 sensitivity (IC50)
High
Low
MRS
***
Tacedinaline sensitivity (IC50)
High
Low
MRS
***
SB505124 sensitivity (IC50)
High
Low
MRS
C
**
AZD 5438 sensitivity (IC50)
High
Low
MRS
*
Niraparib sensitivity (IC50)
High
Low
MRS
*
RSL-3 sensitivity (IC50)
High
Low
MRS
**
Erastin sensitivity (IC50)
High
Low
MRS

STable 1. Correlations between the MRS and the infiltration levels of the critical immune cells

	CIBERSORT	XCELL	MCPCOUNTER	QUANTISEQ	EPIC	ImmuCELLAI
**B cell**	NULL<br>B cell naïve: -0.22<br>B cell memory: 0.25<br>B cell plasma: -0.44	0.21<br>B cell naïve: -0.12<br>B cell memory: 0.18	0.25	0.27	0.16	0.14
**CD8+ T cell**	NS	0.23<br>CD8+ T cell naïve: NS<br>CD8+ T cell central memory: 0.25<br>CD8+ T cell effector memory: 0.10	0.14	0.23	-0.36	0.12
**CD4+ T cell**	CD4+ T.cell naive: NULL<br>CD4+ T.cell memory resting: NS<br>CD4+ T cell memory activated: NS	CD4+ T cell naive: 0.20<br>CD4+ T cell memory: 0.12<br>CD4+ T cell central memory: NS<br>CD4+ T cell effector memory: 0.28<br>CD4+ T cell non regulatory: 0.11	NULL	0.1	0.32	0.3
**Neutrophil**	-0.13	-0.10	-0.19	-0.24	NULL	-0.27
**Macrophage**	Macrophage: NS<br>Macrophage M1: 0.13<br>Macrophage M2: 0.43	Macrophage: 0.45<br>Macrophage M1: 0.50<br>Macrophage M2: 0.40	NS	Macrophage: NULL<br>Macrophage M1: NS<br>Macrophage M2: NS	0.55	0.55
**Dendritic Cell**	Myeloid DC: NULL<br>Myeloid DC resting: NS<br>Myeloid DC activated: NS	Myeloid DC: 0.37<br>Myeloid DC activated: 0.46<br>Plasmacytoid DC: NS	Myeloid DC: 0.2	Myeloid DC: 0.16	NULL	Myeloid DC: 0.46
**Monocyte**	NS	0.12	NS	NS	NULL	0.12
**NK**	NK resting: -0.10<br>NK activated: 0.12	NS	NS	NS	NS	0.12
**Cancer associated fibroblast**	NULL	NS	NS	NULL	0.4	NULL

Supplementary Figure Legends

SFigure 1. (A-C) Consensus clustering distribution function (CDF), area under CDF curve increment, and tracking plot for k = 2 to 9. (D) MRS was positively correlated with age, Gleason score (GS) and TNM stage, but not with PSA levels. Student's t test; *, $p < 0.05$; **, $p < 0.01$; ***, $p < 0.001$.

SFigure 2. Validation of the MRM in Taylor and ICGC PCa cohorts. (A) The distribution of risk scores in Taylor and ICGC PCa database based on median of MRS. Blue represents low MRS subgroup, while red represents high MRS subgroup. (B) The distribution of disease free patients(blue) or recurred (red) in subgroups. (C) The heatmap of five constituent genes of MRM, MRM characteristics and clinical features in Taylor and ICGC PCa database.

SFigure 3. Validation of the MRM in real world samples using qRT-PCR. (A) Heatmap of the relative mRNA expression of five constituent genes of MRM in PCa patients (n = 30) based on the results of qRT-PCR. (B) MRS was calculated based on the relative expression of these genes. Scatter plots showed the relationship between MRS and clinical features.

SFigure 4. (A) Top10 mutated genes ordered by mutation rate in different MRS subgroups of TCGA PCa samples. Color coding indicates mutation type. The top shows the total number of mutations, while the right shows the percentage of mutations. (B) Amino acids-related gene sets enriched in low-MRS subgroup.

SFigure 5. Immune characteristics of different MRS subgroups. (A) B cells- and T cells-related gene sets enriched in high MRS subgroup ($p < 0.05$, $FDR < 0.25$). (B) Stroma-related gene sets enriched in high MRS subgroup ($p < 0.05$, $FDR < 0.25$). (C) The proportions of TME cells in different MRS subgroups based on six independent algorithms (CIBERSORT, XCELL, QUANTISEQ, MCPCOUNTER, EPIC and ImmuCELLAI). (D) Predicting response to immunotherapy in TCGA PCa samples using the online tool TIDE.

SFigure 6. Validation the MRM for predicting drug sensitivity based on Taylor cohort. (A) Estimated sensitivity of current clinically preferred drugs for advanced PCa

in Taylor cohort patients with high and low MRS risk. (B-C) Predicting sensitivity of potential drugs for advanced PCa in Taylor cohort patients with high and low MRS risk.
